# Primary skeletal muscle cells from chronic kidney disease patients retain hallmarks of cachexia *in vitro*

**DOI:** 10.1002/jcsm.12802

**Published:** 2022-01-14

**Authors:** Luke A. Baker, Thomas F. O'Sullivan, Katherine A. Robinson, Matthew P.M. Graham‐Brown, Rupert W. Major, Robert U. Ashford, Alice C. Smith, Andrew Philp, Emma L. Watson

**Affiliations:** ^1^ Department of Health Sciences University of Leicester Leicester UK; ^2^ Department of Respiratory Sciences University of Leicester Leicester UK; ^3^ John Walls Renal Unit University Hospitals of Leicester NHS Trust Leicester UK; ^4^ Department of Cardiovascular Science NIHR Leicester Cardiovascular Biomedical Research Unit Leicester UK; ^5^ Leicester Orthopaedics University Hospitals of Leicester Leicester UK; ^6^ Department of Cancer Studies University of Leicester Leicester UK; ^7^ Mitochondrial Metabolism and Ageing Laboratory Garvan Institute of Medical Research Sydney NSW Australia; ^8^ St Vincent's Clinical School UNSW Medicine, UNSW Sydney NSW Australia; ^9^ Department of Cardiovascular Sciences University of Leicester Leicester UK

**Keywords:** Skeletal muscle, Cachexia, Chronic kidney disease, Protein breakdown, Anabolic resistance

## Abstract

**Background:**

Skeletal muscle wasting and dysfunction are common characteristics noted in people who suffer from chronic kidney disease (CKD). The mechanisms by which this occurs are complex, and although progress has been made, the key underpinning mechanisms are not yet fully elucidated. With work to date primarily conducted in nephrectomy‐based animal models, translational capacity to our patient population has been challenging. This could be overcome if rationale developing work could be conducted in human based models with greater translational capacity. This could be achieved using cells derived from patient biopsies, if they retain phenotypic traits noted *in vivo*.

**Methods:**

Here, we performed a systematic characterization of CKD derived muscle cells (CKD; *n* = 10; age: 54.40 ± 15.53 years; eGFR: 22.25 ± 13.22 ml/min/1.73 m^2^) in comparison with matched controls (CON; n = 10; age: 58.66 ± 14.74 years; eGFR: 85.81 ± 8.09 ml/min/1.73 m^2^ ). Harvested human derived muscle cells (HDMCs) were taken through proliferative and differentiation phases and investigated in the context of myogenic progression, inflammation, protein synthesis, and protein breakdown. Follow up investigations exposed HDMC myotubes from each donor type to 0, 0.4, and 100 nM of IGF‐1 in order to investigate any differences in anabolic resistance.

**Results:**

Harvested human derived muscle cells isolated from CKD patients displayed higher rates of protein degradation (*P* = 0.044) alongside elevated expression of both TRIM63 (2.28‐fold higher, *P* = 0.054) and fbox32 (6.4‐fold higher, *P* < 0.001) in comparison with CONs. No differences were noted in rates of protein synthesis under basal conditions (*P* > 0.05); however, CKD derived cells displayed a significant degree of anabolic resistance in response to IGF‐1 stimulation (both doses) in comparison with matched CONs (0.4 nm: *P* < 0.001; 100 nM: *P* < 0.001).

**Conclusions:**

In summary, we report for the first time that HDMCs isolated from people suffering from CKD display key hallmarks of the well documented *in vivo* phenotype. Not only do these findings provide further mechanistic insight into CKD specific cachexia, but they also demonstrate this is a reliable and suitable model in which to perform targeted experiments to begin to develop novel therapeutic strategies targeting the CKD associated decline in skeletal muscle mass and function.

## Introduction

Skeletal muscle wasting and dysfunction is a common characteristic of chronic kidney disease (CKD).^1^ This can limit physical activity, resulting in a downward spiral of atrophy, deconditioning and disuse, leading to poorer outcomes.^2^, ^3^ These are important clinical problems as they negatively impact upon quality of life/life participation, increase rates of morbidity and mortality and subsequently the continued rise of health and social care costs worldwide. The mechanisms underpinning muscle wasting and dysfunction are not yet completely elucidated in human CKD.^4^ To date, this important clinical question has largely been investigated using either animal models of CKD,^5^ which do not always replicate what we see in our CKD patients, or human muscle biopsies,^2^, ^3^, ^6^, ^7^ which while physiologically relevant, represent a single time point and are limited with regard to the investigation of the dynamic mechanisms involved in such processes. Pinpointing the mechanisms of such a phenotype is key in order to design appropriate therapeutic interventions in people with CKD. As such, the development of a biologically relevant model of CKD human skeletal muscle is important in order to enable more detailed investigations to develop our understanding of muscle loss and dysfunction in human CKD.

Research seeking to depict the mechanisms of muscle wasting in CKD populations has proven it to be complicated and multi factorial, as expected in this metabolically compromised population.^4^ There is a general consensus that the loss of muscle mass and function noted in CKD patients is due to elevations in protein degradation,^8^, ^9^, ^10^ as opposed to reduction in synthesis, leading to a negative protein balance and consequential loss of mass.^4^ Although still unconfirmed, recent research suggests that reductions in protein synthesis does occur in this populations once End Stage Kidney Disease (ESKD) is reached.^11^, ^12^ To this point, research has directed its efforts at depicting the mechanisms contributing to protein breakdown in this population, such as metabolic acidosis,^13^, ^14^ chronic inflammation,^15^, ^16^, ^17^, ^18^ satellite cell dysfunction,^12^, ^19^ and more recently the role of microRNAs.^5^, ^20^

Skeletal muscle models of atrophy are not uncommon, with recent work systematically defining a high through put test bed for the screening of therapeutic interventions.^21^ Studies which have sought to model the characteristics of CKD skeletal muscle *in vitro* have looked to manipulate the proposed systemic environment of CKD and expose this environment to immortalised cells lines.^16^, ^22^ Although this has proven effective, it fails to represent the phenotypic adaptation of CKD muscle, characteristics that could only be replicated through the use of tissue derived from the patients themselves. One key paper^16^ reported exciting findings highlighting the potential for CKD to drive protein degradation via a Toll‐like receptor‐4 (TLR4) dependent mechanism, suggesting this as a potential therapeutic target. However, findings here were in found in immortalised cell‐lines (C2C12) in response to CKD derived serum, making findings hard to translate due to the lack of CKD derived cellular phenotype and the genomic translational differences noted between species. Human derived muscle cells (HDMCs), which is a term used to encompass the multicellular population harvested from a skeletal muscle biopsy tissue, have been shown to retain their phenotypic traits, including disturbances in metabolic processes.^23^ Therefore, we sought to develop a primary culture model established from muscle biopsies collected from patients with advanced non‐dialysis dependent CKD in which more rigorous experimentation can be performed. Skeletal muscle primary cells are increasingly used to research muscle disorders which is a feature of other chronic illnesses,^24^, ^25^, ^26^, ^27^ but are yet to be utilized to investigate muscle wasting in human CKD. Such a model would provide an opportunity for mechanistic investigations specific to this disease state, enabling progression of the current knowledge base and the development of CKD specific therapeutic interventions, with the underpinning rational for translation into patient populations. The aim of the current investigation was to elucidate the phenotypic traits which are maintained in CKD derived cells compared with matched control (CON) cells in order to provide a novel model for the study of uraemic cachexia as well as provide insight into potential mechanisms by which this may occur *in vivo*.

## Materials and methods

### Patient recruitment

Biopsy samples collected from both populations in this investigation were recruited under the Explore CKD study (ISRCTN: 18221837). All patients were recruited from nephrology outpatient clinics at Leicester General Hospital, UK. All matched controls (CON) were recruited from orthopaedic outpatient clinics at Leicester General Hospital who were admitted for unrelated purposes. Exclusion criteria were age <18 years, pregnancy, any disability that prevented patients from undertaking exercise, insufficient command of English, or an inability to give informed consent. Ethical approval was given by the National Research Ethics Committee (15/EM/0467). All patients gave written informed consent, and the trial was conducted in accordance with the Declaration of Helsinki.

### Isolation and subsequent culture of human derived muscle cells

Skeletal muscle samples were obtained from non‐dialysis CKD (CKD) patients (*n* = 10) utilizing the micro biopsy technique and samples from CON (n = 10) (Demographics displayed in *Table* 1) were obtained via theatre dissection methods as previously reported.^7^ Once skeletal muscle tissue was obtained, specimens were visually inspected and connective tissue removed before undergoing a digestion method. Briefly, muscle biopsies were mechanically dissociated using a sterile scalpel blade and scissors, prior to two enzymatic digestions (collagenase IV [1 mg/mL], BSA [5 mg/mL], trypsin [500 μL/mL]) of 20 and 15 min at 37°C. The resulting supernatant was filtered through a 70 μm nylon filter and centrifuged for 5 min at 800 *g*. The pellet was washed with HamsF10 (1% PS, 1% gentamycin) and plated on uncoated 9 cm^2^ petri dish for 3 h in order to separate myogenic and non‐myogenic cells. The cell suspension was transferred to 25 cm^2^ flask coated with collagen I to facilitate adhesion of myogenic cells, which once proliferated in Growth medium (GM: HamsF10, 20% FBS, 1% PS, 1% AMP) through at least one proliferative cycle, cells were then taken forward for experimentation. At the first passage HDMC cultures were taken through immunocytochemistry analysis (as detailed below) for the calculation of Desmin positivity, to provide an insight into the isolated cellular population. Combined Desmin positivity of all isolated populations was calculated at 68.2% ± 5.4 and 65.3% ± 8.2 at passage one for CON and CKD cultures (respectively).

**Table 1:** Donor biopsy demographic details

Characteristic	Match controls (CON) (*n* = 10)	CKD patients (CKD) (*n* = 10)
Age (years)	58.66 ± 14.74	54.40 ± 15.53
Gender (men/women)	2/8	3/7
eGFR (mL/min/1.73 m^2^)	85.81 ± 8.09	22.25 ± 13.22
Ethnicity (%), White British	100	100

### Experimental design

The current investigation used two experimental designs to first conduct initial phenotyping of HDMCs derived from CKD patients compared with CONs, with the second phase of experiments allowing for the investigation into how myotubes derived from each donor type responded to IGF‐1 stimulation. For both experiments, cells isolated from digestion protocols were seeded into collagen coated flasks and proliferated to generate the required cell numbers for experimental work. All cells were taken through at least one cell passage prior to experimentation. Once cell numbers were sufficient, cells were plated into 6‐well plates at 100 000 cells per well and proliferated for 3–5 days to achieve confluence in GM. Once confluence was achieved, cells were switch to a low serum medium for 7 days to induce differentiation (DM: DMEM, 2% HS, 1% PS). Samples were taken at confluence (D0), 3 days into differentiation (D3) and 7 days into differentiation (D7).

For the second phase of experimentation, the culture process was identical to the previous experiments until D7, at which point cells were washed three times in 1 mL HBSS prior to 2‐hour stimulation with either (i) control (0 nm); (ii) low (0.4 nm); or (iii) high (100 nm) dose of IGF‐1 (Thermo Scientific, UK, 100 μg, MW; 7649 Da) in 1 mL serum free DM. At the end of the 2 h, cells were harvested for western blot analysis.

### Ribonucleic Acid extraction and Polymerase Chain Reaction experiments

Total RNA was extracted from both skeletal muscle tissue (10 mg wet weight) and primary cells using Trizol® (Invitrogen, UK) and 1 μg RNA was reverse transcribed to cDNA using an AMV reverse transcription system (Promega, Madison, WI, USA). Primers, probes and internal controls for all genes were supplied as Taqman gene expression assays (Applied Biosystems, Warrington, UK) as follows: Ki‐67 (Hs00242962_m1); Pax7 (Hs00242962_m1); MyoD (Hs02330075_g1); Myf5 (Hs00929416); Myogenin (Hs010722232_m1); MyHC1 (Hs00428600_m1); MyHC2 (Hs00430042_m1); MyHC3 (Hs01074230_m1); MyHC7 (Hs01110632_m1); MyHC8 (Hs00267293_m1); IL‐6 (Hs00985639_m1); TNFα (Hs01113624_g1); Myostatin (Hs00976237_m1); TRIM63 (Hs00822397_m1); Fbxo32 (Hs00369714_m1) with 18 s (Hs99999901_s1) as an internal control. All reactions were carried out in a 20 μL volume, 1 μL cDNA, 10 μL 2× Taqman Mastermix, 8 μL water, 1 μL primer/probe on an Agilent Biosystem Light Cycler with the following conditions, 95°C 15 s, followed by 40× at 95°C for 15 s and 60°C for 1 min. The Ct values from the target gene were normalized to 18 s and expression levels calculated according to 2^−ΔCt^ method to determine fold changes.

### Western blotting

At the designated time point, cells were washed once in 1× PBS and subsequently scraped in 400 μL of RIPA buffer (Sigma Aldrich, UK) supplemented with Phosphatase Inhibitor Cocktail (Sigma Aldrich, UK, P0044) and centrifuged at 800 *g*. The resulting supernatant was collected, and protein concentration determined using the Bio‐Rad DC protein assay. Lysates containing 30 μg protein were subjected to SDS‐PAGE using 10% stain free gels (Bio‐Rad, UK) on a mini‐protean tetra system (Bio‐Rad, UK). Once ran, gels were activated for 45 s prior to transfer, the subsequent post activation image was used for densitometry analysis for normalization of protein load. After proteins were transferred onto nitrocellulose membranes (unless otherwise stated), membranes were blocked for 1 h with tris‐buffered saline with 5% (*w*/*v*) skimmed milk powder and 0.1% (*v*/v) tween‐20 detergent. Membranes were incubated with the primary antibody overnight. Antibodies to determine p‐Akt^ser473^ (Cell Signalling, UK; 4060), t‐Akt (Cell Signalling, UK; 9272), puromycin (ThermoFisher Scientific, UK; A10685) were used at 1:1000 dilution in tris‐buffered saline with 2% skimmed milk powder. Following washing, membranes were incubated with species specific horseradish peroxides conjugated secondary antibodies and visualized using EZ‐chemiluminescence detection kit (Geneflow, Lichfield, UK). Band intensity was captured using ChemiDoc touch instrument (BioRad, UK) and quantified using Image Lab Software (BioRad, UK).

### Protein degradation

Cellular proteins were pre‐labelled with 2uci/mo L‐[3H]‐Phenylalanine for 3–4 days prior to experimentation. At the end of this period cells were washed in HBSS and test media (TM) added that contained 2 mmol/L unlabelled phenylalanine to minimize reincorporation of unlabelled L‐Phe into cellular protein. After the first 2 h of TM incubation, media was discarded and replaced with fresh TM in order to eliminate any effect of rapidly degraded protein on overall protein degradation rates, this was designated as time zero. Protein degradation rates were calculated from the rate of release of 14C into the cellular medium, which was sampled after 9, 24, and 48 h. Rates are expressed as log10 of the percentage of the initial cellular 3H per hour.

### Protein synthesis

Assessment of protein synthesis was made using the Surface Sensing of Translation (SUnSET) assay. This involves immunodetection of puromycin during peptide elongation as a measure of protein synthesis.^28^ For this analysis, modifications were made in the experimental phase of tissue culture. Cells were incubated with 100 μm puromycin (ThermoFisher Scientific, UK) 30 min prior to cellular harvest. The process for the detection and quantification of puromycin was conducted via western blotting techniques similar to that described earlier, but samples were transferred onto polyvinylidene fluoride membranes (PDVF) activated with 100% methanol as opposed to nitrocellulose membranes.

### Immunocytochemistry

Cells were fixed in 4% paraformaldehyde for 20 min at room temperature, washed three times with PBS and then blocked and permeabilized in PBS containing 5% goat serum and 0.25% Triton X‐100 for 1 h. Cells were incubated with rabbit anti‐desmin primary antibody (1/400; Cell Signalling, UK) at 4°C overnight, washed three times in PBS, and incubated with Alexa Flour 488‐labelled goat anti‐rabbit IgG (1/400; ThermoFisher Scientific, UK) for 2 h at room temperature protected from the light. DAPI (100 ng/mL; ThermoFisher Scientific, UK) was used to visualize the nuclei. Five images were taken per well using a FLoid imaging system (ThermoFisher Scientific, UK) with images being taken using a systematic approach with the first image being taken in the centre of the well and the subsequent four images taken at the 12, 3, 6, and 9 o'clock positions in order to provide a true (represented in the Supporting Information, *Data* S1), unbiased representation of the primary cultures. Once images were acquired, images analysed using Fiji (v2.1.0). Myotube diameter was assessed at three points on each cell and myotube tube number determined per image frame. Fusion indexes were defined by the number of DAPI positive nuclei within myotubes (desmin positive cell containing three or more nuclei) divided by the total number of DAPI positive nuclei. Desmin positivity analysis was conducted on cell populations derived from each donor at the earliest possible passage to give an indication of myogenic percentage, this was defined as the number of DAPI positive nuclei incorporated into all desmin positive cells divided by the total number of DAPI positive nuclei.

### Statistical analysis

Data are presented as mean ± SD unless otherwise stated with individual donors displayed to indicate variation. Statistical analyses were performed using SPSS v.25 (IBM, Chicago, IL, US). Data were tested for normal distribution and homogeneity of variance. Analysis of differences across time and condition were analysed using 2‐way repeated measures analysis of variance (ANOVA), with Bonferroni post‐hoc analysis used in order to detect where interaction effect lay within the data set. Non‐parametric equivalents were used where appropriate. Significance was assumed at *P* ≤ 0.05 with raw values overlayed for the presentation of donor‐donor variation, unless otherwise stated.

## Results

### Myogenic progression

**Figure 1:**
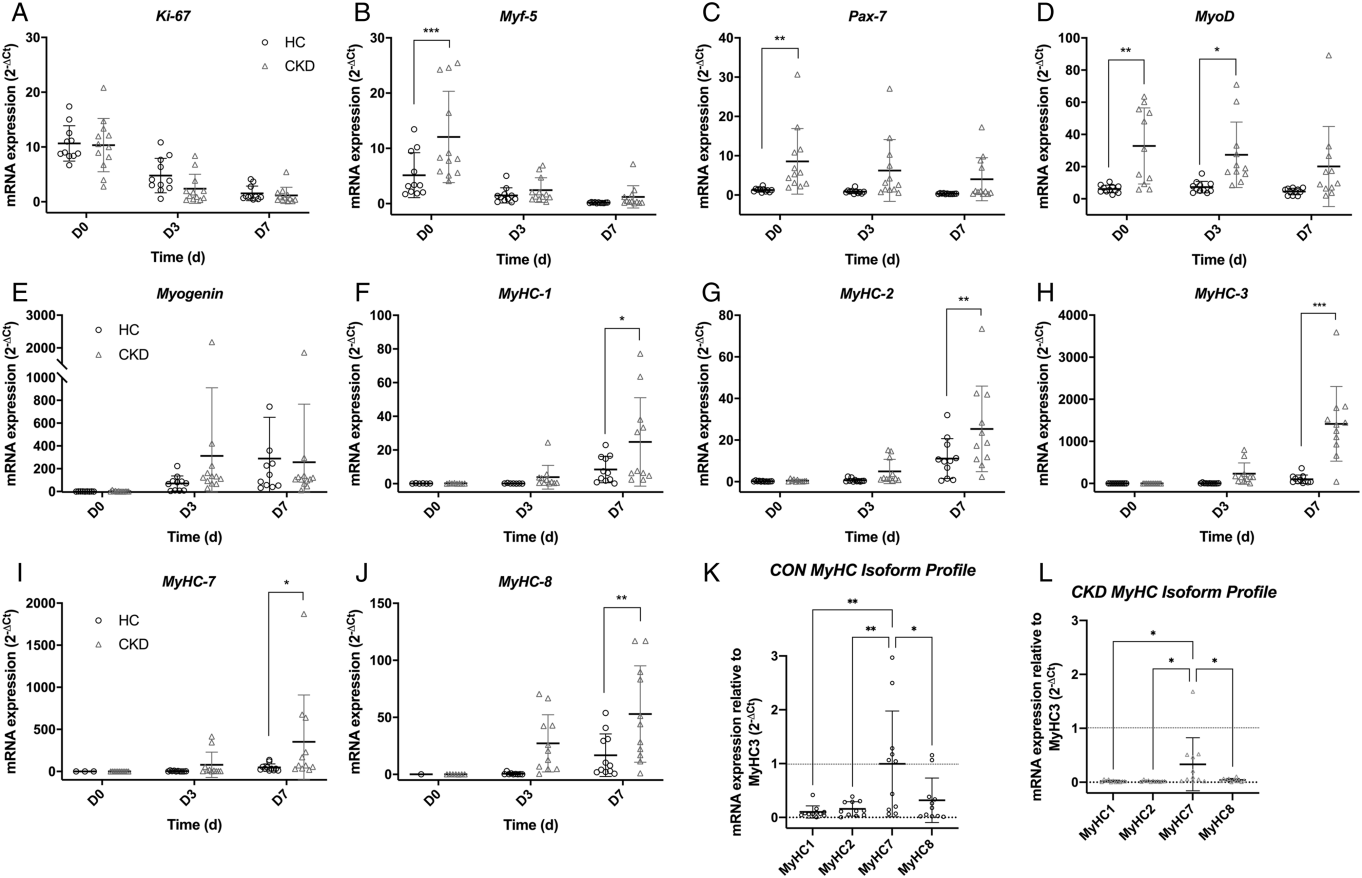
Time course comparison between muscle derived cells from CKD patients and matched controls. mRNA gene expression was quantified at days 0, 3, and 7 of the experimental time courses for the following markers: *(A)* Ki‐67, *(B)* Myf‐5, *(C)* Pax7, *(D)* MyoD, *(E)* Myogenin, *(F)* MyHC‐1, *(G)* MyHC‐2, *(H)* MyHC‐3, *(I)* MyHC‐7, *(J)* MyHC‐8, *(K)* MyHC isoform profiles for CON derived cells at D7. *(I)* MyHC isoform profile for CKD derived cells at D7. Data depicted as individual repeats with an overlay of mean ± SD. *P ≤ 0.05; **P ≤ 0.01; ***P ≤ 0.001. Data displayed includes *n* = 4 CON and *n* = 4 CKD donors across 12 experimental repeats

Gene expression analysis was performed in order to determine if the skeletal muscle cell growth cycle differed between donor groups (CKD vs. CON). At the point of confluence (pre‐differentiation, D0), markers of proliferation (Ki‐67, Myf‐5) were noted to be highly expressed in both cell populations with no differences seen in Ki‐67 mRNA expression, but significantly higher expression of Myf‐5 seen in cells derived from CKD patients (*P* = 0.0004). Further to this, at D0 both Pax‐7 and MyoD expressions were significantly greater in CKD derived cells at the earliest time point (*P* = 0.005 and *P* = 0.001, respectively), with significantly higher levels held through to D3 in regard to MyoD (*P* = 0.034). Regarding myotube maturity, a panel of myosin heavy chain (MyHC) markers were quantified. At the latest time point (D7), the relative expression of all MyHCs were noted to be significantly higher in cells derived from CKD patients (MyHC‐1 *P* = 0.013; MyHC‐2 *P* = 0.003; MyHC‐3 *P* < 0.001; MyHC‐7 *P* = 0.022; MyHC‐8 *P* = 0.005). When MyHCs were normalized to MyHC‐3 (which is the most immature of the MyHC isoforms) to indicate MyHC profile ratios, similar profiles were noted in both cell types, with the greatest contribution being noted from MyHC‐7 in both CON (*P* < 0.001) and CKD (*P* < 0.001) cells, followed by MyHC‐8 in CON myotubes (*Figure* 1).

### Cellular inflammatory status

**Figure 2:**
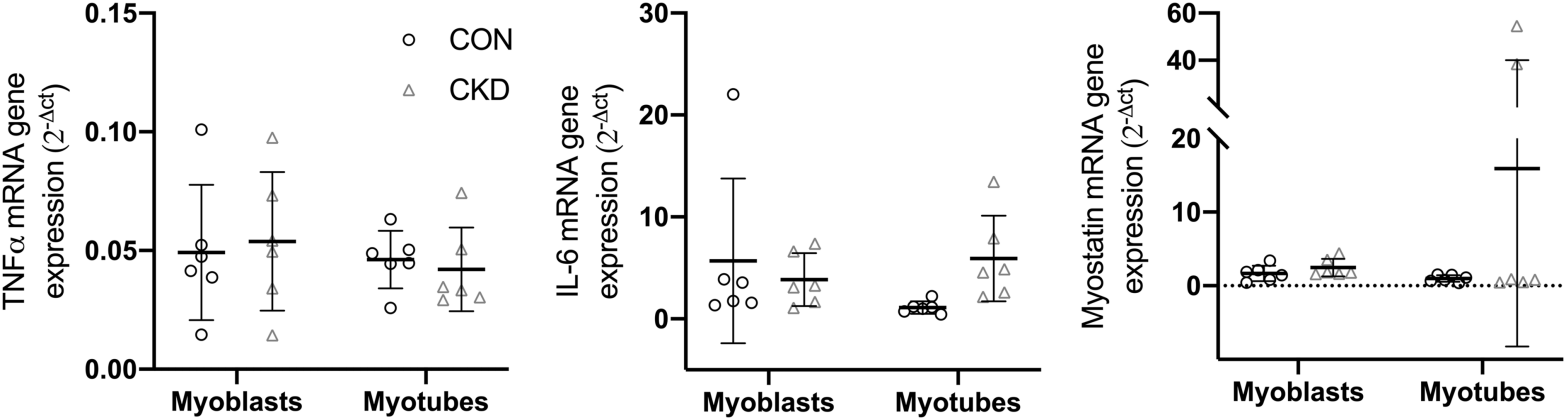
mRNA expression analysis of inflammatory cytokines TNFa, IL‐6, and myostatin in muscle‐derived cells from both CKD and CON donors. Markers were quantified in both myoblasts (D0) and myotubes (D7). Data depicted as individual repeats presented with an overlay of mean ± SD. Data displayed includes *n* = 3 CON and *n* = 3 CKD donors across 6 experimental repeats

Inflammatory gene expression analysis was conducted in both myoblasts and myotube cultures from cells derived from CKD and CON donors. No significant differences were noted between either population for expression of IL‐6, TNFα or Myostatin in either myoblasts (D0) or myotubes (D7) (all *P* > 0.05) (*Figure* 2).

### Protein breakdown

**Figure 3:**
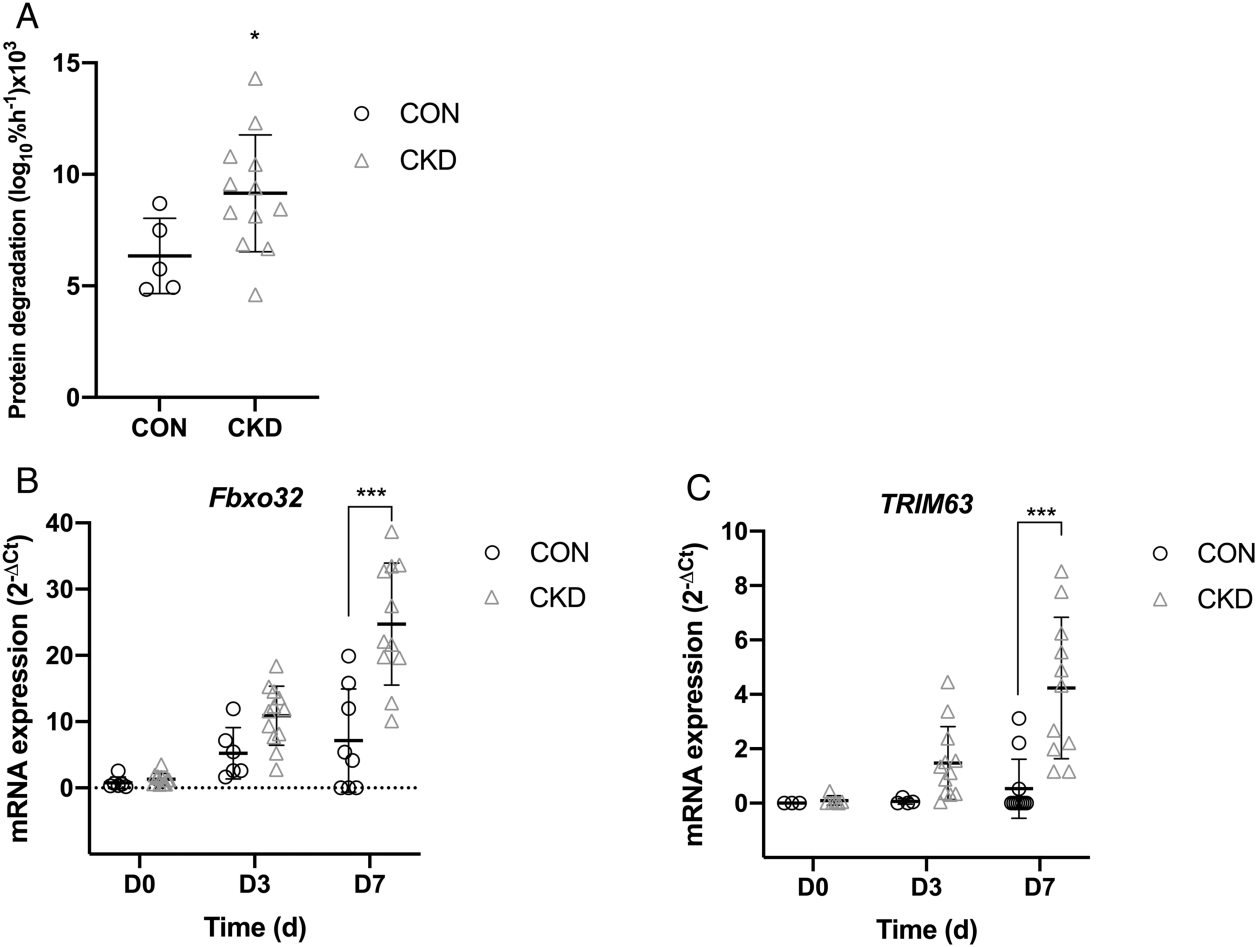
*(A)* protein degradation rates measured in myotubes (D7) derived from both CKD and CONs. *(B, C)* mRNA gene expression analysis of markers of protein breakdown Fbxo32 and TRIM 63 across a 7 day time course in both CKD and CON derived cells. Data presented as individual data points with an overlay of mean ± SD. *P ≤ 0.05, ***P ≤ 0.001. Data presented from *n* = 3 CON and *n* = 4 CKD across *n* = 18 experimental repeats

Protein degradation rates were significantly higher in myotubes (D7) derived from CKD donors in comparison with CON derived cells (*P* = 0.044, *Figure* 3A). Following analysis of mRNA gene expression markers related to protein breakdown, higher expression of fbxo32 was noted in CKD derived myotubes (*Figure* 3B, D7) in comparison with those from CONs (*P* < 0.001). Similar trends were also noted for TRIM63 (*Figure* 3C), with near significant elevations being noted in CKD derived cells in comparison with CON (*P* = 0.054) at day 7. No further differences were noted in either fbxo32 or TRIM63 at D0 and D3.

### Protein synthesis

**Figure 4:**
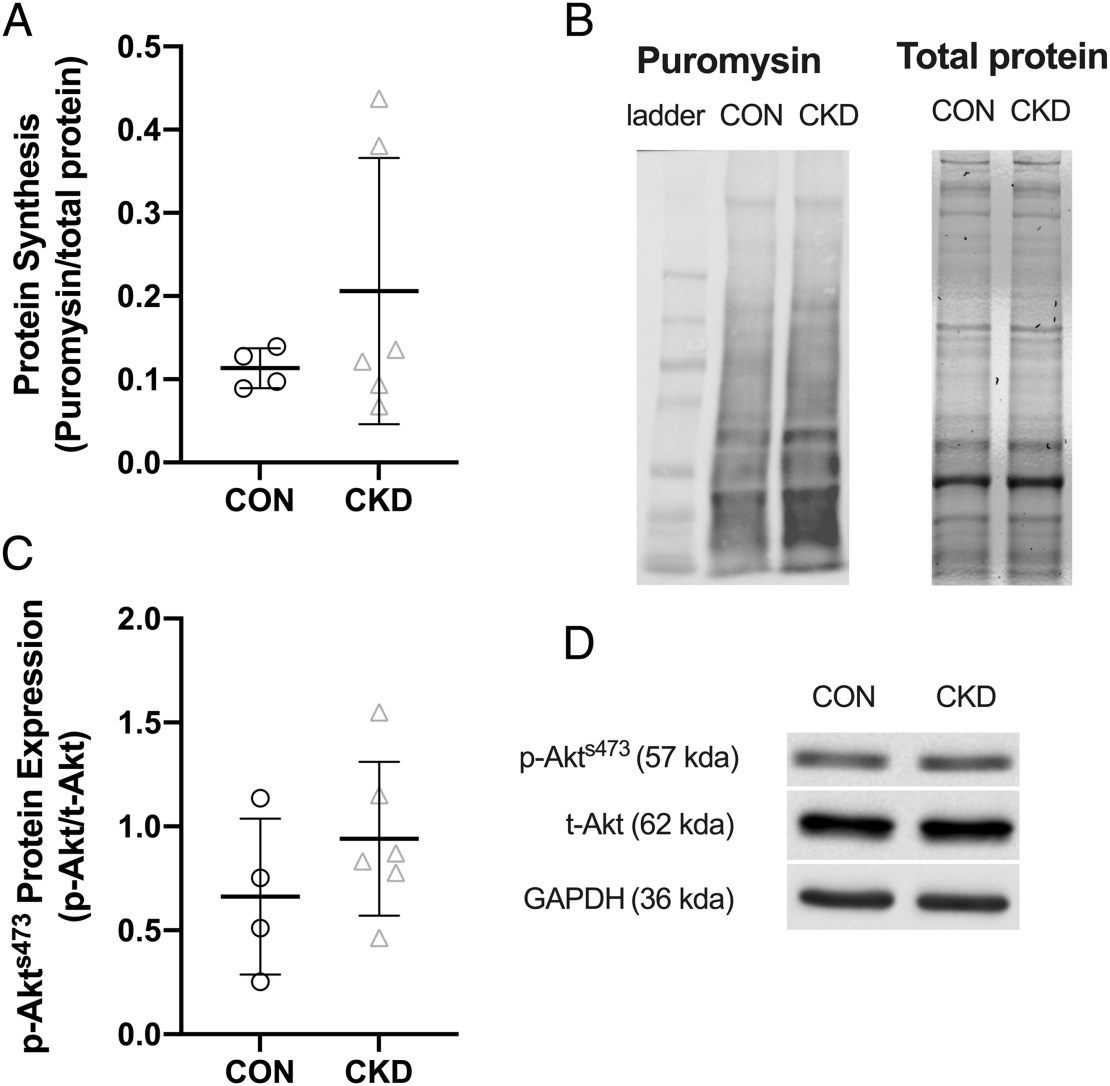
*(A)* Protein synthesis measured by SUnSET assay in both CON and CKD derived myotubes (D7). *(B)* Representative densitometry images for puromycin incorporation and total protein content. *(C)* Densitometry analysis of p‐Akt made relative to t‐Akt in both CON and CKD derived myotubes. *(D)* Representative densitometry images for the quantification of p‐Akt^S473^. Data presented as individual data points with an overlay of mean ± SD. Data presented from *n* = 3 CON and *n* = 4 CKD across *n* = 10 experimental repeats

Protein synthesis measures were taken in basal conditions (i.e. with cells in GM) in fully differentiated myotubes (D7). No significant differences were found in protein synthesis rates between cells from CKD and CON donors (*P* = 0.29) (*Figure* 4A). To further investigate protein synthesis, we quantified p‐Akt expression as a common marker used to indicate changes in protein synthesis at the signalling level (*Figure* 4B). Here, we again noted no differences between the two cell types (*P* = 0.66).

### Morphology

**Figure 5:**
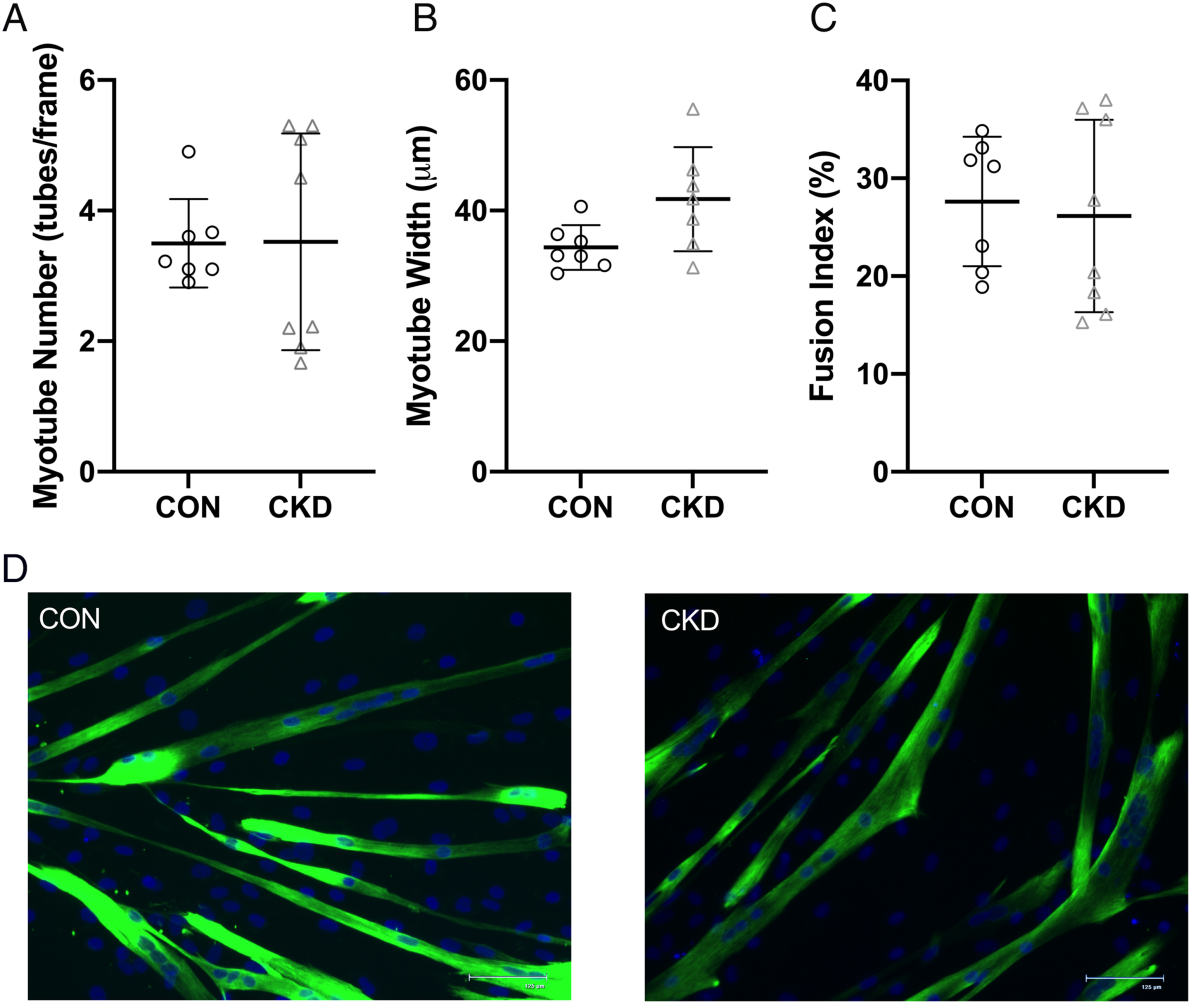
Morphological analysis of myotubes (D7) derived from CON and CKD cells. *(A)* Myotube number per image frame; *(B)* myotube width; *(C)* fusion index as a percentage of nuclei within differentiated myotubes; *(D)* representative images of both CON and CKD myotubes. Staining displays DAPI (blue) and DESMIN (green). Data presented as individual data points with an overlay of means ± SD. Data presented from *n* = 3 CON and *n* = 4 CKD across *n* = 15 experimental repeats

With data to this point suggesting CKD derived cells inherently display elevated protein degradation rates and no differences in protein synthesis, we sought to investigate if elevations in protein degradation translated to a morphological change. On morphological analysis of myotubes (D7) from cells derived from CON and CKD patients, we saw no differences in myotube, number (*Figure* 5A; *P* > 0.05), diameter (*Figure* 5B; *P* > 0.05) or fusion index (*Figure* 5C; *P* > 0.05). Representative images of both conditions can be visualized in *Figure* 5D.

### IGF‐1 response

**Figure 6:**
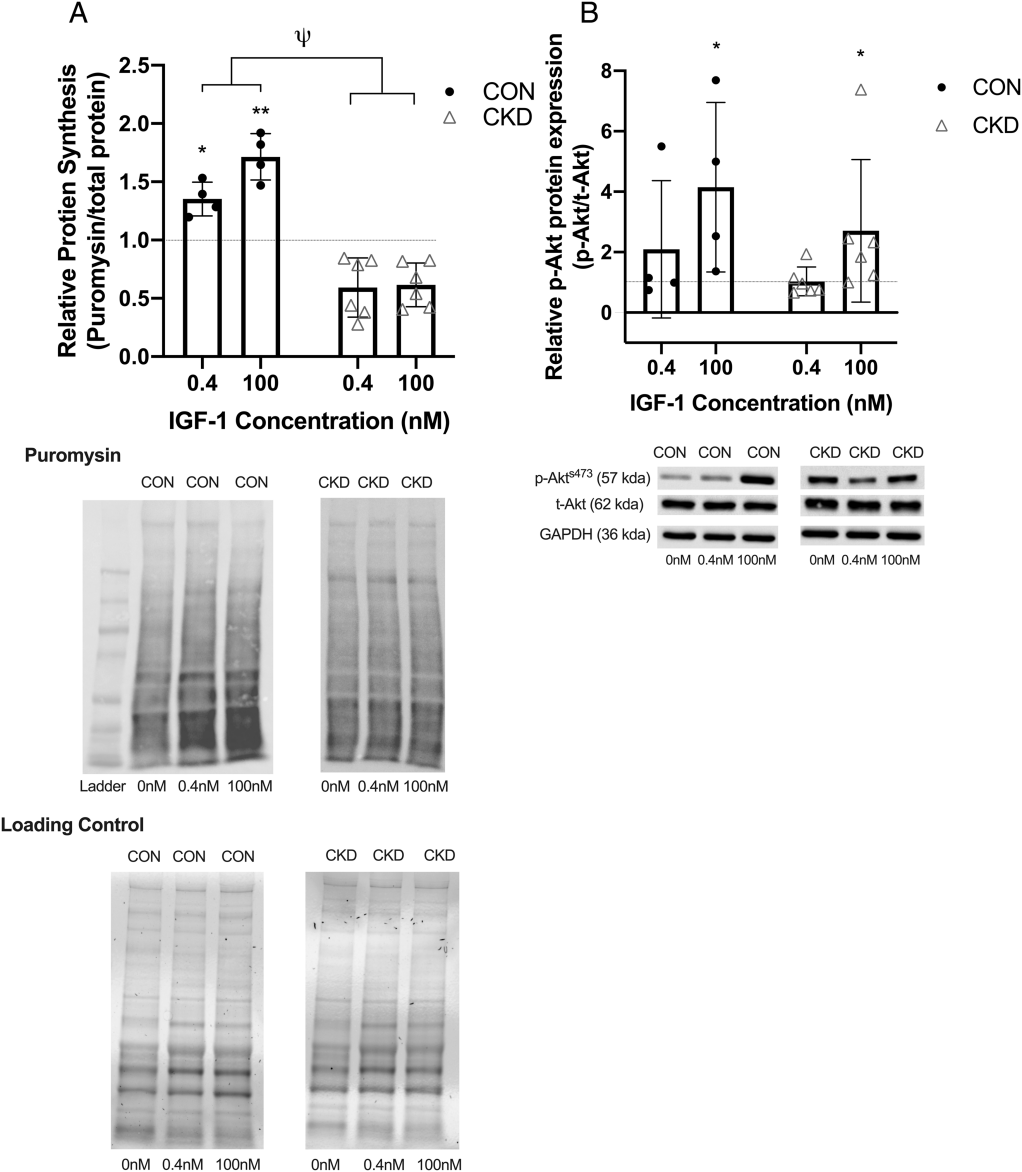
Indicators of protein synthesis in response to the exposure of CON and CKD derived myotubes to either 0.4 or 100 nM of IGF‐1 for 2 h. *(A)* Quantification of SuNSET assay as a measures of protein synthesis. *(B)* Representative densitometry images for puromyosin and the loading controls for the quantification of protein synthesis. *(C)* Quantification of p‐Akt^s473^. *(D)* Representative densitometry images for p‐Akt^S473^, t‐Akt and GAPDH for the quantification of p‐Akt^s473^. Data displayed relative to the 0 nM control condition and as individual data points with an overlay of mean ± SD. *P ≤ 0.05 between the indicated condition and control; **P ≤ 0.01 between the indicated condition and control; ^ψ^ *P* ≤ 0.001 main effect between donor groups. Data presented from *n* = 2 CON and *n* = 3 CKD across *n* = 10 experimental repeats

With anabolic resistance commonly being reported as a phenotypic trait of CKD muscle, our final set of experiments sought to investigate whether IGF‐1 induced protein synthesis and Akt phosphorylation was affected by cell donor origin. Both 0.4 nM (*P* = 0.012) and 100 nM (*P* < 0.001) IGF‐1 stimulated significant increases in protein synthesis in a dose dependent manner in CON derived myotubes (*Figure* 6A). However, in CKD derived myotubes, no significant differences were observed in protein synthesis in response to either dose of IGF‐1 (*P* = 0.21). We also quantified p‐Akt expression as an indicator of the activation of anabolic related signalling (*Figure* 6B). Here, we saw significant elevations in p‐Akt in both the CON (*P* = 0.025) and CKD derived myotubes (*P* = 0.041) in response to the 100 nM dose of IGF‐1 (*Figure* 6B), with no significant differences noted in either donor group in response to the lower concentration or between the two donors (*P* = 0.265).

## Discussion

Understanding the causal mechanisms that underpin uraemic cachexia is key for the development of therapeutic interventions to combat this debilitating co‐morbidity in people with kidney disease. Research conducted within this patient population has shown that uraemic cachexia manifests early in the disease process^29^ and as such, targeting patients in stages G1–4 may allow for a preventative approach to management.^30^ Progress in understanding such uraemic skeletal muscle cachexia has predominantly been conducted in patient populations,^2^, ^3^, ^6^, ^7^, ^31^ with any cellular work to this point using immortalised cells lines.^16^, ^22^ Even though work has begun to utilize serum from CKD patients in order to investigate the role of the uraemic environment,^16^ this work is limited in translational capacity due to a lack of a fully characterized *in vitro* model of human CKD skeletal muscle that retains a uraemic phenotype. As such we report for the first time a comparison of CKD and matched CON derived primary skeletal muscle cells. This work both defines the CKD derived cellular population for use as a future test bed for investigation, as well as describes the phenotypic differences between the populations.

When investigating changes in muscle mass and function in any population it is important to first understand its primary presentation, whether that be elevations or reductions in protein synthesis, protein breakdown, or a combination of the two. In the CKD population, *in vivo* research predominantly suggests that muscle loss is primarily due to elevations in protein degradation,^1^, ^10^ as opposed to a reduction in the rates of protein synthesis. Interestingly we found this reported phenotype to be retained *in vitro*, with significant elevations in labelled L‐[3H]‐Phenylalanine release from CKD derived myotubes, indicating elevations in the rate of protein breakdown compared with CON. This was coupled with higher mRNA expression of Fbxo32 and elevated expression of TRIM63.

The role of the translated proteins for both Fbxo32 (MafBx) and TRIM63 (Murf‐1) have been studied at length within CKD populations.^2^, ^16^ As part of the E3‐ligase family, both ligases operate by tagging their targets with ubiquitin (Ub) for subsequent protein breakdown by the proteasome. More specifically, Murf‐1 is involved in the targeted breakdown of sarcomere proteins^32^ including troponin^33^ along with both Myosin Light Chains^34^ and MyHCs.^35^ As such, within an *in vivo* skeletal muscle niche where degradation of such proteins is occurring, it would be expected that elevations in Murf‐1 would lead to reductions in MYHCs. In contrast, here we report elevations in the mRNA expression of both Murf‐1 and MyHCs in CKD derived myotubes. Although not in the same context, previous studies have reported elevated expression of MYHCs in myotubes alongside elevations in protein degradation as we observe,^36^ attributing such results to myotubes having to constantly transcribe new MyHCs in order to offset elevations in the breakdown of sarcomeric based proteins. We believe a similar process may be occurring in our CKD myotubes, with elevations in MyHC expression and cellular process initiated to restore proteostasis. The notion that targeted breakdown of sarcomeric proteins is somewhat supported with the lack of off set in our direct measures of protein synthesis, however any compensatory increases in synthetic rate could also be masked by a potential inhibition in protein synthesis which has been described previously,^11^, ^37^ although not in human based models. Follow up analysis of the translated protein levels in CKD myotubes is required, along with dynamic measures of protein synthetic rate to confirm or deny this hypothesis.

Elevations in MafBx have been shown to regulate the half‐life of both MyoD and downstream eIF3f,^38^, ^39^ leading to the inhibition of myogenesis or downstream protein synthesis. Although we report a significant upregulation in MafBx in CKD compared with CON myotubes, we also report no changes in related measures of protein synthesis or in the expression of MyoD in the same cultures. Furthermore, we found no difference in the basal levels of p‐Akt in CKD derived myotubes compared with CONs. Interestingly, a blunting or suppression of Akt activation in CKD muscle has been reported at length in the literature by multiple groups^40^, ^41^, ^42^ and using immortalised cell lines.^16^ Our data is the first to report that primary skeletal muscle cells taken from CKD patients retain the elevation in basal rates of protein degradation observed *in vivo*, despite not being exposed to circulatory inflammatory signals as would occur. This therefore suggests that muscle from CKD patients may hold an inherent cachectic phenotype, rather than being driven solely by environmental cues, suggesting that this model could be useful in determining the fundamental molecular inducers of cachexia in CKD patients. Interestingly the elevations in protein degradation and related E3‐ligases discussed did not lead to a loss of myotube width. Although surprising, in line with the points raised previously we suggest this to be due to the targeted ubiquitination previously described,^10^ which may lead to a greater decline in function in comparison with mass. This model in its current form is limited in its capacity to measure muscle function in vitro, this is a key contextual outcome when studying uraemic cachexia, and one we hope future experimental advancements will enable us to address.

Markers of proliferation, myogenic potential and myotube maturity were studied in order to determine phenotypic differences in CKD cells compared with CON. Markers of proliferation, notably Myf‐5, were seen to be significantly higher in CKD derived cells. It is well reported that Myf‐5 is required during the regenerative process to allow for the proliferation of myogenic populations (Pax7^+^, which was also shown to be elevated in CKD derived cells) in order to facilitate muscle regeneration^43^ and that this process can be driven by inflammatory stimuli, such as TNF‐α and IL‐6.^43^ Interestingly, here we reported no elevated mRNA expression of inflammatory markers, despite our observed elevations in Myf‐5. Again, we think it is reasonable to suggest that, at least in part, this elevation in Myf‐5 in CKD myoblasts is an epigenetic adaptation, in response to the predisposition of these cells to a uraemic environment, although detailed epigenetic deep dive analysis is required to confirm this. If this were to be the case, this would not only provide an insight in those with uraemic cachexia but would also have mechanistic implications more widely. It should however be highlighted the role of dysfunctional satellite cell populations in sarcopenia is still unclear and warrants further research.^44^, ^45^ Interestingly, differences were also noted in MYHC expression between the two donor groups as discussed earlier. When interpreting MyHC mRNA data, it is important to not only look at the absolute expression, but how the ratios of each MYHC compare to give an indication of culture maturity and MyHC progression. In this regard, relative levels of MyHC7 were seen to be the highest in both cultures, which has been shown to be the dominant lineage of choice previously in human derived myotubes.^46^ We also report trends to reduced MYHC7 in CKD myotubes suggesting a potential inhibition of MyHC maturity in this donor group. As MyHC maturity is highly dependent on the localized niche and mechanical strain,^47^ future work utilizing a highly specialized tissue engineered model of CKD muscle is required to confirm or reject such a hypothesis.

Anabolic resistance has been reported at length in CKD populations, with research identifying attenuated IGF‐1 related signalling^48^, ^49^, ^50^ as well as reduced anabolic signalling in response to exercise.^3^ Our final experiments sought to see if such inhibition was retained *in vitro*. Here we report novel findings showing CKD derived myotubes have significantly reduced levels of protein synthesis in response to IGF‐1 stimulation, at both supramaximal and biologically relevant doses, alongside reductions in p‐Akt^S473^, although these were only significant at supramaximal doses of IGF‐1. These findings in isolation give us the initial indication that the anabolic resistance reported *in vivo* is maintained *in vitro,* providing us with a valuable tool for investigating molecular inducers of cachexia in CKD patients.

To conclude, our findings, for the first time, provide a detailed phenotypic characterization of primary skeletal muscle cells obtained from non‐dialysis dependent CKD patients in comparison with age matched controls (CON). We provide novel data suggesting that the cachectic CKD phenotype reported in vivo is displayed in HDMC cultures in vitro, suggesting that an inherent defect could be present in CKD muscle cells that leads to aberrant growth in both the resting and anabolic states. This observation provides us with a novel tool to enable screening of novel therapeutics to counteract CKD cachexia and determine the molecular inducers of this disorder. Future work should seek to follow up on the mechanistic insights uncovered, as well as address current limitations by performing an *in vivo* cachexic characterization of the recruited population and an ex vivo phenotyping prior to *in vitro* investigation, which will not only be vital for future epigenetic investigations, but potentially uncover new opportunities for the development of personalized medicine approaches for those with uraemic cachexia. This holistic approach will also provide further clarity on whether the phenotype of what are essentially myotubes derived from satellite cells that we report on here, also extends to that of in vivo myofibres. Given that cachexia is thought to impact 50% of patients developing CKD,^51^ tools to develop therapeutic strategies to prevent CKD cachexia are vital to our understanding and management of this debilitating disease.

## Conflict of interest

The authors report no conflict of interest. The authors of this manuscript certify that they comply with the ethical guidelines for authorship and publishing in the *Journal of Cachexia, Sarcopenia and Muscle*.

## Funding

We are grateful to the Stoneygate Trust for their generous financial support of this work. Dr Emma Watson was supported by Kidney Research UK (PDF2/2015). Dr Major was funded by Kidney Research UK (TF2/2015).

## Supporting information


**Data S1.** Supporting information.
