# Supplementary material for: Primary skeletal muscle cells from chronic kidney disease patients retain hallmarks of cachexia in vitro
Source: J Cachexia Sarcopenia Muscle. 2022 Jan 14;13(2):1238–49. doi: 10.1002/jcsm.12802 (PMC8978027; doi:10.1002/jcsm.12802)

**Supplementary Material**

Supplementary Table 1. Peptamen dosing and nutritional information among evaluable patients

| **Patient** | **Peptamen dose** | **Diet** |
| --- | --- | --- |
| **A** | 50 mL/hour for 12-16 hours/day | Tube feeding was supplemented with three daily meals of solid food, snacks in the morning, afternoon, and evening. |
| **B** | 60 mL/hour (C1), increased to 75 mL/hour C2-C3 | Tube feeding was supplemented with three daily meals of solid food. E.g., for breakfast, the patient ate, “orange, 1 slice eggs , scrambled 1/2 egg 1/2 slice whole wheat toast diet gingerale, 2 cans throughout day,” while for lunch, the patient ate,” spanish rice, 1/2 cup refried beans, 1/4 cup cheese enchilada, 2 tbsp 1 slice orange.” The patient consumed an afternoon snack of 6 chips, then ate a dinner that consisted of, “potaotes, roasted 1/2 cup olive oil, 1 tsp cauliflower, 1/2 cup cooked 1 slice orange.” |
| **C** | 60 mL/hour (C1) increased to 70 mL/hour C2-C3 | Tube feeding was supplemented meals of solid food, but the meals were inconsistent according to the 24-hour dietary recall. In Cycle 1, the patient ate breakfast, an afternoon snack, lunch, and dinner. In Cycle 2, the patient ate a morning snack, dinner, and an evening snack. In Cycle 3, the patient ate breakfast, dinner, and an evening snack. |
| **D** | 55 mL/hour | Minimal supplementation with solid food. Patient could not eat. |
| **E** | 40 mL/hour for 10 hours/day (C1), increased to 85 mL/hour for 13.2 hours (C2-C3) | The patient ate three daily meals of solid food, and consumed a morning, afternoon, and evening snack during Cycle 2. |
| **F** | 45mL/hour | Did not complete 24 hour food recall. |
| **G** | 60 mL/hour for 20 hours/day (C1-C2), 70 mL/hour (C3) increased during C3 to 75 mL/day for 16/hours per da | In Cycle 1, the patient did not consume any meals of solid food, and the dietician noted that the, “subject only getting tube feeding NPO.” In Cycle 2, the patient was on a liquid diet, and ate a morning snack (“1/2 cherry popsicle”) and an afternoon snack (“2 oz. jello, black cherry”). |
| **H** | 50 mL/day for 20 hours/day | The patient did not eat daily solid meals during the three cycles, as reported by the 24-hour dietary recall. The patient was on a full liquid diet all three cycles, eating “coffee, reg. 1/2 cup creamer half n half, 1 tsp french vanilla, 1 tsp” for breakfast in Cycle 2. During Cycle 1, the patient ate breakfast, a morning snack, and an afternoon snack. During Cycle 2, the patient only ate breakfast. During Cycle 3, the patient ate breakfast and an afternoon snack. |
| **I** | 55 mL/hour for 18 hours/day (C1)  70 mL/hour for 14 hours/day (C2-C3) | The patient ate breakfast and lunch in Cycle 1 and 3. The patient had a morning snack, afternoon snack, dinner and an evening snack in Cycle 1. The patient was tube feeding only during Cycle 2 and 4 and did not eat solid foods. The patient is on insulin. |
| **J** | 60 mL/hour (C1) increased to 80 mL/hour for 18 hours/day (C2-C3) | The patient was consuming a clear liquid diet during Cycle 1 and was tube feeding only in Cycle 2. During Cycle 1, the patient ate a “strawberry popsicle” for breakfast, “apple juice” as a morning snack, “fruit punch 6 oz x 3” for their afternoon snack, and “clear chicken soup 8 oz” for dinner. |
| **K** | 65 mL/hour for 23 hours/day | The patient was only getting tube feeding NPO, and did not eat solid foods |
| **L** | 40 mL/hour for 24 hours/day (C1) increased to 50 mL/hour in C2 and 60 mL/hour in C3 for 21 hours/day | The patient was tube feeding only with some liquids in Cycle 2. The patient ate breakfast during Cycle 1 and 3. The patient had a morning snack in Cycle 2 of “cherry gatorade, 6 oz.,” an afternoon snack of “blue gatorade, 6 oz,”and an evening snack of “purple gatorade, 6oz.” The patient only ate lunch and dinner during Cycle 3. The patient experienced vomiting and bloating that was possibly related to the intervention. |
| **M** | 80 mL/hour for 8 hours/day (C1), 80 mL/hour for 22 hours/day (C2-C3) | In addition to the tube feeding, the patient ate regular solid food meals. During Cycle 1, the patient ate breakfast, a morning snack, lunch, an afternoon snack, and dinner. During Cycle 2, the patient ate breakfast, lunch, dinner, and an evening snack. The patient experienced hypophosphatemia and hypokalemia that was possibly related. C3 diet information missing. |
| **N** | 75 mL/hour for 17 hours/day (C1), decreased to 75 mL/hour for 13 hours/day in C2, and 100 mL/hour for 13 hours/day in C3. | The patient is vegetarian. During Cycle 1, the patient ate breakfast, lunch, an afternoon snack, and dinner. During Cycle 2, the patient ate an afternoon snack, and dinner. The patient said “she didn’t feel well-maybe had the flu so did not eat very much.” During Cycle 3, the patient ate breakfast, lunch, an afternoon snack, and dinner. |
| **O** | 85 mL/hour of Peptamen (C1) | Missing 24 hour recall data. |
| **P** | 50 mL/hour for 15 hours/day (C1) | Supplemented with solid foods including breakfast, lunch and dinner for Cycles 1-3. Also takes creon shakes with every meal and protein shakes 1-2 times per day. Does not eat raw vegetables. |

Supplementary Figure 1. Change in appendicular lean mass by cycle


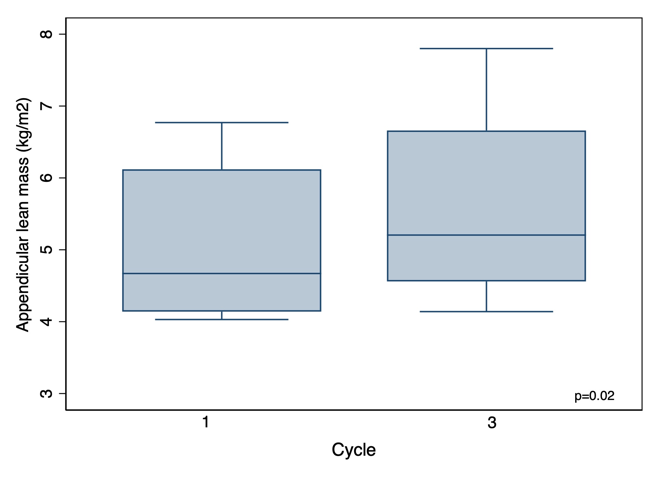


Supplementary Figure 2. Health related quality of life (EORTC QLQC30) subscale scores by cycle


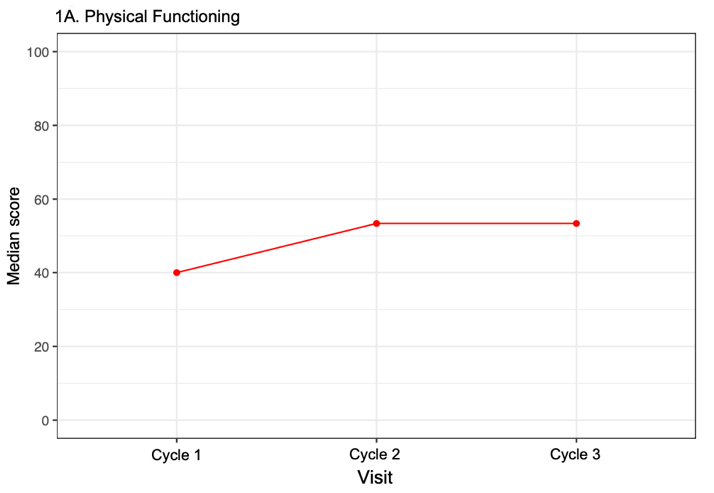

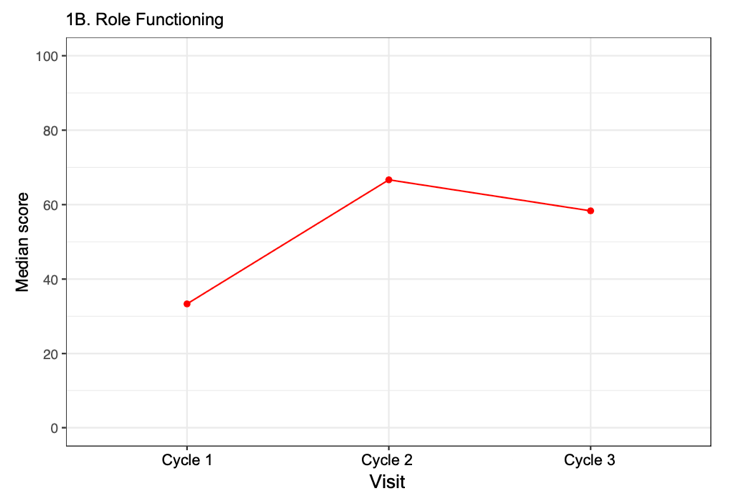


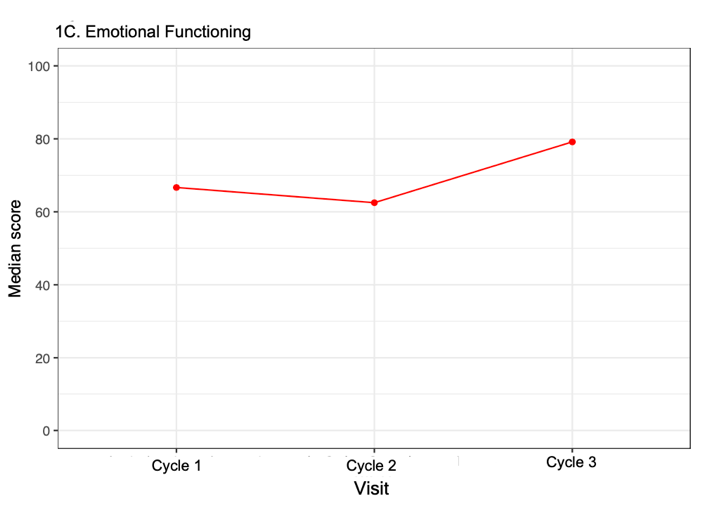

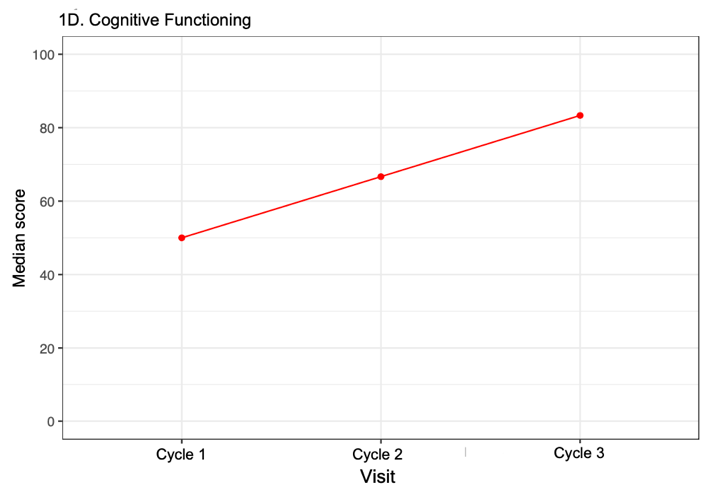


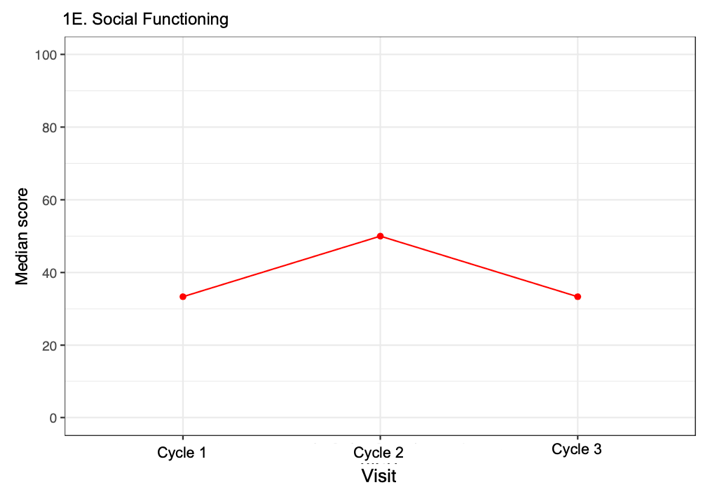

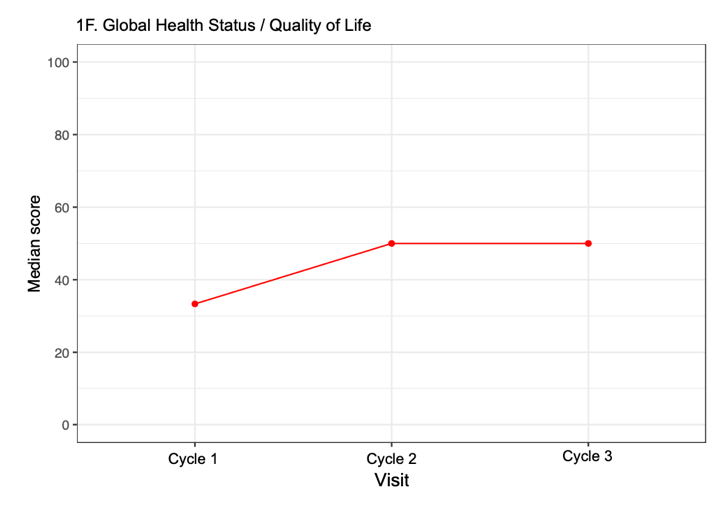


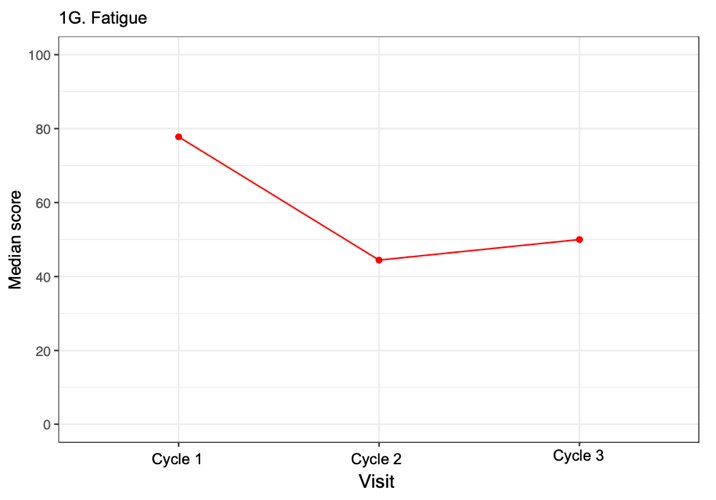

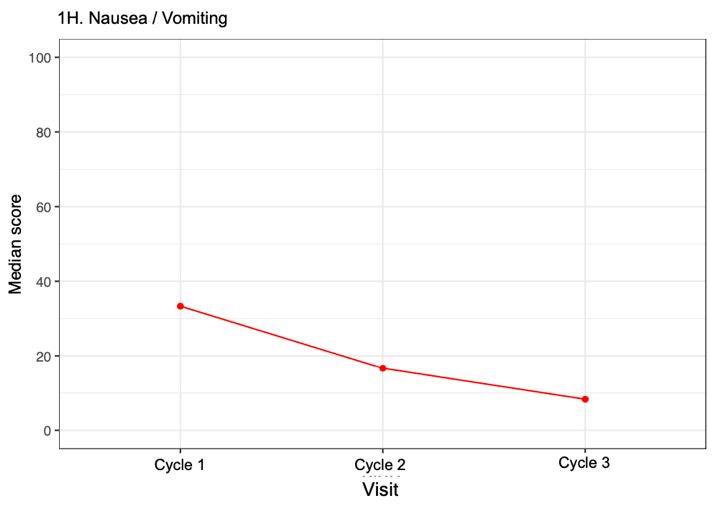


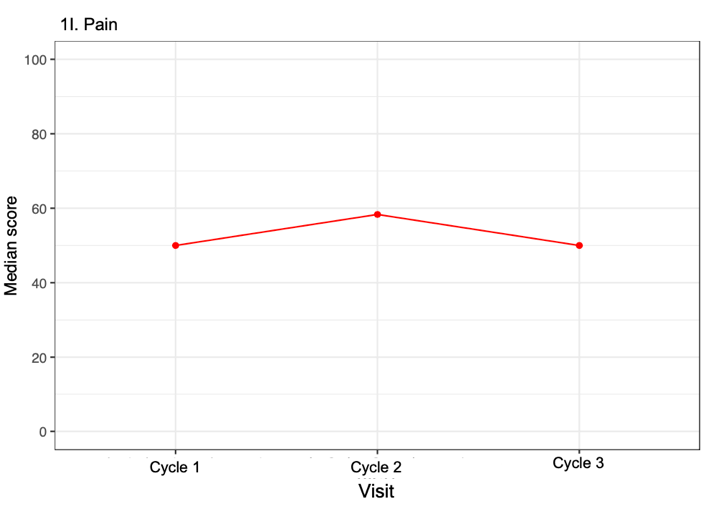

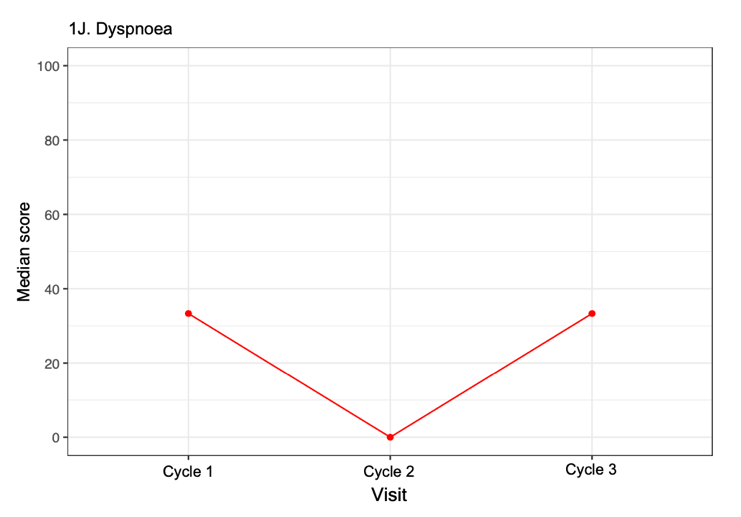


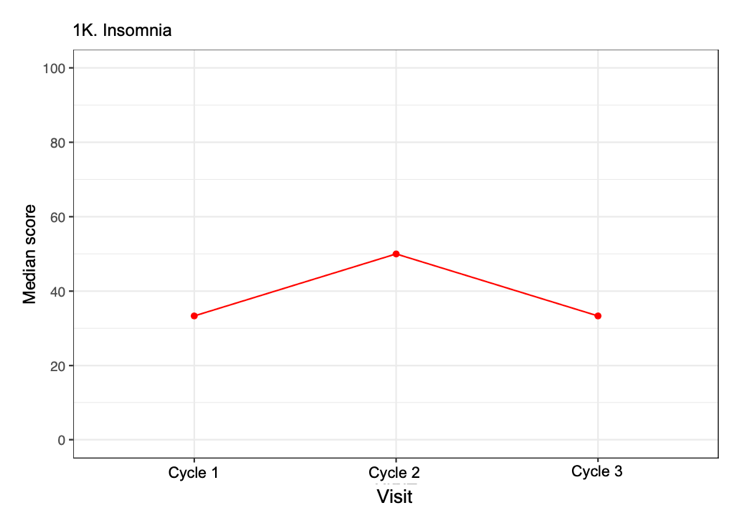

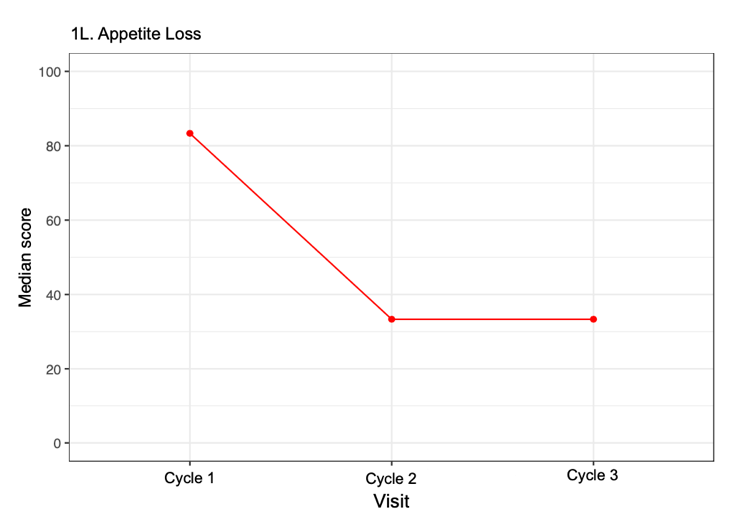


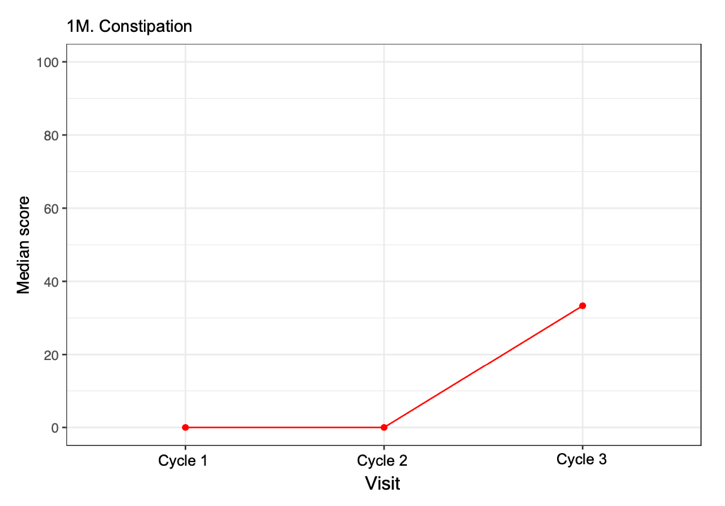

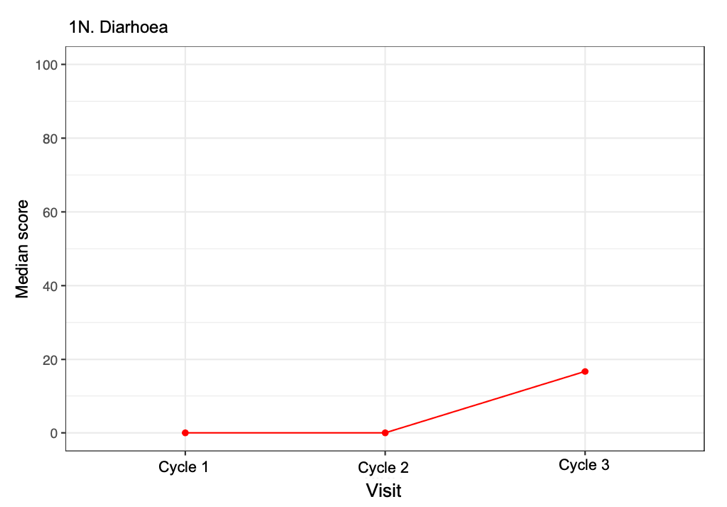


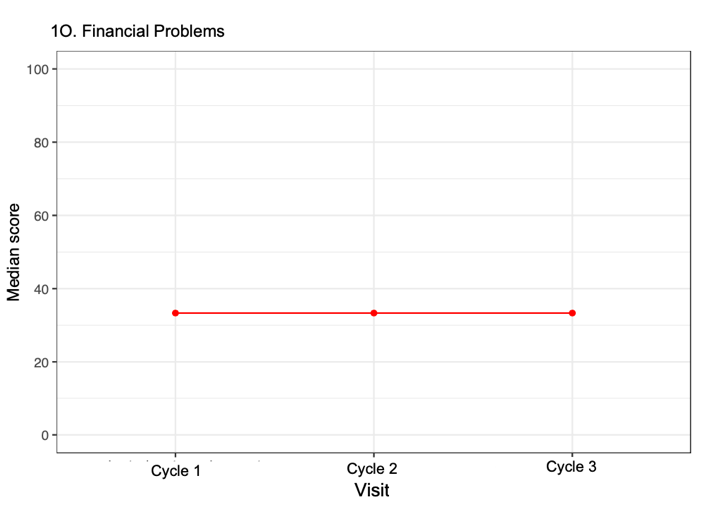

Supplement: Supplementary file 1 — Data S1. Supporting information. [file JCSM-13-1238-s001.docx]
